# Effect of muscle energy technique on pain, importance of physical activity, self-confidence, and posture in COPD patients with chronic neck pain: An experimental randomized controlled study

**DOI:** 10.1097/MD.0000000000042849

**Published:** 2025-06-20

**Authors:** Serpil Mihçioğlu, Mehtap Malkoç

**Affiliations:** a Department of Physiotherapy and Rehabilitation, Faculty of Health Sciences, Eastern Mediterranean University, Mersin, Turkey.

**Keywords:** COPD, muscle energy technique, physical activity, posture, self-confidence

## Abstract

**Background:**

Excessive use of accessory respiratory muscles leads to postural abnormalities and pain in the cervical area in patients with chronic obstructive pulmonary disease (COPD). This study aimed to evaluate the effects of the muscle energy technique (MET) on pain, importance of physical activity (PA), self-confidence, and posture in patients with moderate COPD and chronic neck pain.

**Methods:**

This study is a randomized controlled parallel-group trial with an experimental design that includes a longitudinal follow-up process. This study included 52 COPD patients (26 in the MET group) (mean forced expiratory volume in one second [FEV1%: 63.53 ± 9.33] and 26 in the control group [CG] [FEV1%: 64.06 ± 8.53]). The MET group received MET 3/week for 4 weeks. Both groups participated in a home-based exercise program. Pain intensity (visual analog scale), importance of PA (importance scale), self-confidence (confidence scale), and posture (New York Posture Rating Chart) of all individuals were evaluated before and after the 4-week treatment period, as well as at the end of the 6-week follow-up period.

**Results:**

Significant improvements were observed in both groups (*P* < .05). However, while a notable reduction in pain intensity was observed in the intervention group, significantly greater improvements were recorded in the perception of the importance of exercise, self-confidence in exercising, and postural alignment (*P* < .05). At week 4, pain data for both groups (resting, activity, and night pain scores, respectively) were as follows: low level pain (MET group: 25 ± 96.2, 26 ± 61.9, 26 ± 100.0; CG: 13 ± 50, 16 ± 38.1, 16 ± 61.5) and moderate level pain (MET group: 1 ± 3.8, 0, 0; CG: 13 ± 50, 10 ± 100.0, 10 ± 38.5). Additionally, the effects on the perception of exercise importance, self-confidence, and posture were maintained during the follow-up period (*P* < .05).

**Conclusion:**

MET performed on the accessory respiratory muscles improved pain intensity and the importance of PA, confidence, and posture. MET was found to provide superior responses compared to the control group. Therefore, MET should be included as an important part of treatment programs for COPD patients with chronic neck pain.

## 1. Introduction

Chronic obstructive pulmonary disease (COPD) is one of the most significant chronic inflammatory lung diseases with increasing mortality and morbidity worldwide. Globally, the prevalence of COPD is increasing and is estimated to account for 5.4 million deaths in 2060.^[1]^

In COPD patients, pulmonary hyperinflation leads to respiratory muscle alterations by diminishing diaphragm contraction. The workload is shifted to the accessory respiratory muscles, which increases airway resistance and limits the airflow.^[2]^

In patients with COPD, lung hyperinflation, chest wall rigidity, and increased workload on accessory respiratory muscles lead to postural disturbances.^[3]^ Postural changes, such as increased anterior tilt of the head, protraction of the scapula, increased cervical lordosis, decreased mobility of the thoracic spine, and increased lumbar lordosis, result in spasms and pain in the cervical area.^[4]^

Pain is a common extrapulmonary feature in patients with COPD, with a prevalence ranging from 21% to 82%.^[5,6]^ Increased pain severity has been associated with increased dyspnea, decreased exercise capacity, poorer quality of life, and a greater number of specific comorbidities.^[6,7]^ Thus, pain may contribute to poor clinical outcomes.^[8]^

Pain associated with postural impairment and extra load on the spine significantly affects daily activities.^[9]^ Due to the low level of daily physical activity (PA) in individuals with COPD, strategies to increase PA, including exercise training, pharmacological management, and PA counseling, have been implemented, yet have been reported to have limited success in this population.^[10,11]^ Exercise habits are an important factor affecting PA levels in individuals with COPD. Exercise habits, especially in previously undiagnosed patients, are associated with PA and disease deterioration.^[12]^

People who are not physically active lack the confidence to be active, even if they know that it is essential for their health.^[13]^ The reasons for this lack of self-confidence are health-related concerns. A lack of confidence was defined as low self-efficacy.^[14]^ Although COPD patients are physically able to perform activities, they avoid routine activities of daily living due to low self-efficacy.^[15,16]^ Among patients with COPD, self-efficacy has been found to be positively associated with PA.^[17]^ It is extremely important to develop individualized care interventions to improve the self-efficacy of individuals with COPD.^[18,19]^

It has also been shown that some passive treatments reduce pain and have a place in the management of patients with chronic pain.^[20,21]^ Recent guidelines for the management of chronic neck pain suggest multimodal care such as stress self-management, high-dose massage, mobilization, manipulation, soft tissue therapy, supervised group exercise, supervised yoga, supervised strengthening exercises, or home exercises.^[22–25]^

Manual therapeutic methods are increasingly preferred for the treatment of COPD.^[26]^ Manual therapy includes soft tissue therapy, joint manipulation, and mobilization and has the potential to assess variations in respiratory mechanics related to decreased lung function. It is also used to manage musculoskeletal pain and increase the mobility of the chest area by reducing dyspnea.^[27]^ Muscle energy technique (MET) is a manual therapy technique used to improve joint mobility, stretch tense muscles and fascia, improve circulation, and reduce pain.^[28]^ This technique is considered an effective method for pain management and increasing range of motion by causing muscle stretching followed by relaxation after contraction^.[29]^

The studies emphasize the importance of treatment approaches aimed at reducing the negative effects of pain on clinical outcomes.^[30–32]^ These findings suggest that pain management and the correction of postural abnormalities should be considered as part of the treatment process in individuals with COPD. Additionally, according to studies such as those by Lee et al and Hansen et al., which investigate the relationship between pain and activities of daily living, pain obstructs the physical functions and rehabilitation processes of individuals with COPD.^[6,33]^ Langer et al also emphasized that pain frequently interferes with a patient’s ability to focus and their capacity to participate in respiratory rehabilitation programs.^[34]^ In this context, our study highlights the importance of pain management and assessment, emphasizing the need to strengthen the literature in this field. The aim of this study is to investigate the effects of MET intervention on pain intensity, the importance of PA, self-confidence, and posture in individuals with COPD and chronic neck pain. It is hypothesized that MET intervention will lead to a significant reduction in pain intensity, an increased perceived importance of PA, and improvements in self-confidence and posture in individuals with COPD and chronic neck pain. In this context, it is believed that MET offers a new and different treatment option compared to previously suggested approaches and provides additional insights into this area. This study aims to contribute to the existing literature by examining the effects of pain management and postural corrections in more detail.

## 2. Materials and methods

### 2.1. Study design

This randomized controlled trial and longitudinal study was conducted between March 2021 and February 2022 in the Department of Pulmonology, Gazimağusa/Burhan Nalbantoğlu State Hospital, Turkish Republic of Northern Cyprus, Turkey. This study was approved by the Eastern Mediterranean University Health Ethics Subcommittee (approval date 23.03.2021 and numbered 2021/0066). This study was registered at ClinicalTrials.gov (registration number: NCT04874571). This study was conducted in accordance with the Declaration of Helsinki guidelines.

### 2.2. Determination of the sample size

The sample size was determined using G*Power version 3.1.9.2. The sample size estimation was based on the expected change in pain intensity over time, considering the primary outcome variable as pain intensity. This estimation accounted for the anticipated reduction in pain levels at different time points throughout the study. Considering the statistical tests utilized in the analysis, the initial sample size (α = 0.05, β = 0.80, and Cohen *d* = 0.8) was determined for a total of 52 patients. Due to the possibility of missing cases for various reasons within the scope of the study, the initial sample size was increased by 25%, and the final sample size was determined to be 66 patients.

### 2.3. Participants

A total of 508 patients admitted to the Department of Pulmonology, Gazimağusa and Burhan Nalbantoğlu State Hospitals between March 2021 and February 2022 and diagnosed with COPD by the relevant physician were screened for eligibility criteria. This study was conducted at the Cardiopulmonary Physiotherapy Unit, Faculty of Health Sciences, Eastern Mediterranean University.

Inclusion criteria: Individuals between 35 to 65 years of age, diagnosed with moderate COPD by a pulmonologist according to Gold criteria,^[35]^ neck pain for more than 3 months, and no diagnosis of fibromyalgia.

Exclusion criteria: Patients with COPD exacerbation; those who received physiotherapy for neck pain in the last 3 months; patients with musculoskeletal problems such as disc herniation, fractures, etc in the cervical and thoracic regions; patients who underwent cervical surgery; and participants with medical conditions such as uncontrolled diabetes, hypertension, and severe cardiovascular diseases that prevent safe participation in PA. The use of medications by individuals (e.g., painkillers, aspirin, etc) was not taken into account.

The 64 individuals who volunteered to participate in the study and met the criteria were randomized (minimization) and divided into 2 groups (MET group, n = 32; control group, n = 32). An informed consent form was signed by the individuals who agreed to participate. Written informed consent was obtained from the patient for the publication of the case details and related images included in this case report.

### 2.4. Randomization

The 64 participants who met these criteria were randomly divided into 2 groups using a minimization program running under the DOS operating system. The minimization was based on age, sex, occupation, and pain intensity. The participants were blinded to the group assignment. Participants in the randomization knew whether they would perform exercise, but they did not know whether it was a MET or home exercise. Assessments were performed before treatment, after treatment, and 6 weeks after the end of treatment (follow-up). All evaluations and treatments were performed by the same physiotherapist. The experimenter is aware of which intervention the patients underwent. The participant flow diagram is presented in the Flow Diagram format.

### 2.5. Interventions

All interventions were provided by a physiotherapist with more than 8 years of clinical experience in cardiopulmonary rehabilitation. Individuals in the MET group were included in the MET treatment program and were provided with a home exercise program. Individuals in the control group underwent only a home exercise program.

#### 2.5.1. MET

**Figure 1.**
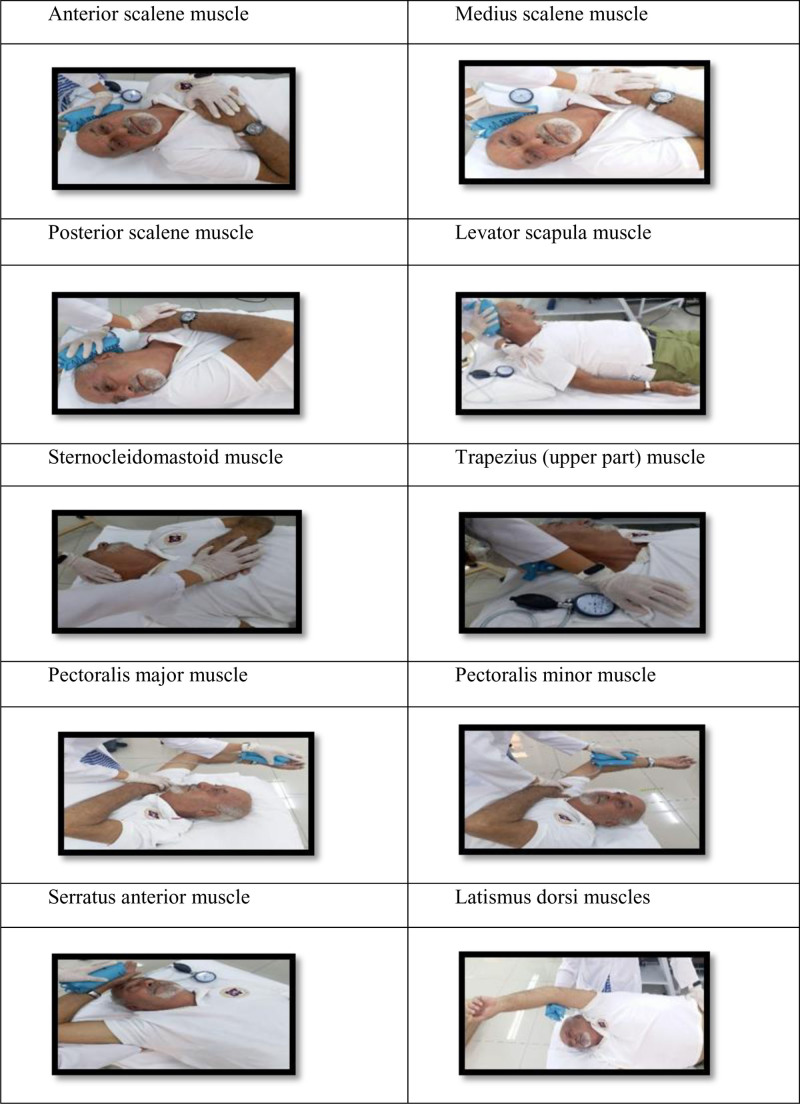
MET training application.

According to Lewit post-isometric relaxation method, MET was applied bilaterally to the scalene (anterior–middle–posterior), levator scapula, sternocleidomastoid, upper trapezius, pectoral muscles, serratus anterior, and latissimus dorsi muscles. MET was performed once for each muscle (each set consisting of 3 repetitions), 3 times a week for 4 weeks, with each session lasting approximately 45 minutes (Fig. 1). The maximum isometric contraction strength of each muscle was measured using a stabilizer pressure biofeedback device (Stabilizer, Chattanooga Group Inc., Hixson). After the patient was properly positioned for each muscle, the measured isometric contraction strength was recorded, and 20% of the obtained value was calculated and used during the treatment.^[36]^ The patient was asked to perform a 7-second isometric contraction against the therapist’s resistance at the point where movement limitation was experienced, corresponding to 20% of the maximum isometric contraction force. The accuracy of the isometric push force was verified using the biofeedback device. After the exercise, the patient was instructed to exhale and relax. Subsequently, the physiotherapist moved the patient’s head to a new barrier point, and the technique was repeated.^[37]^

#### 2.5.2. Home exercise program

A home exercise program including breathing, thoracic expansion, posture, abdominal, stretching, and relaxation exercises training was provided. The patients were instructed using a simple brochure that was illustrated. They were asked to perform the exercise program presented to them for a total of 12 sessions for 4 weeks, every other day, 3 days a week. The exercises lasted approximately 60 minutes. The patients were regularly telephoned on the same day each week. The patients recorded their exercise date during the follow-up program.^[38]^

### 2.6. Measurements of outcomes

The general characteristics of the participants (age, gender, height, weight, body mass index, smoking consumption, duration of smoking, duration of pain, education status, exercise habits, and spirometric measurements) were recorded. All assessments were conducted by the same physiotherapist at baseline (one day before the first treatment session), after 4 weeks (the day after the completion of all treatments), and at the end of the 6-week follow-up.

#### 2.6.1. Pain

The severity of pain at rest, during activity, and at night was assessed using the visual analog scale. The results were recorded in centimeters (cm). Visual analog scale has been reported to be a reliable and valid tool for measuring pain intensity. Pain intensity was categorized as <3 mild pain, 3 to 6 moderate pain, and >6 severe.^[39,40]^

#### 2.6.2. Importance of PA

The importance of PA scale is a single-item measure used in similar studies to assess an individual’s perceived importance of PA. While single-item measures do not allow for traditional internal consistency calculations (e.g., Cronbach alpha), previous studies have demonstrated their test–retest reliability and construct validity in COPD populations.^[41]^ The importance of PA from the patients’ perspective was assessed using an importance scale. They were asked to mark the number on the scale describing how important PA was for them (0 = not at all important and 10 = very important). Patient responses were recorded for statistical analysis.^[18]^

#### 2.6.3. Self-confidence

The self-confidence scale used in this study is based on a self-efficacy assessment method previously validated for individuals with chronic diseases, including COPD. Kara and Mirici validated the Turkish version of the COPD Self-Efficacy Scale and reported a Cronbach alpha value of 0.80 to 0.85, indicating good internal consistency and reliability.^[42]^ Patients’ self-confidence in exercise was assessed using a confidence scale. On this scale, the patient was asked to think about their confidence in performing PA and mark the appropriate number (0 means I have no confidence, and 10 means I have a lot of confidence).^[13]^

#### 2.6.4. Postural assessment

The postures of the patients were assessed using a New York Posture Rating Chart (NYPR). The NYPR consists of a 13-item ranking of body segments (head, neck, shoulder, scapula, upper thoracic spine, waist, rips, abdomen, hips, knees, legs, feet, and toes). Each body segment was scored as 5 (correct posture), 3 (slight deviation), and 1 (pronounced deviation). The total score ranged from 13 to 65. A low score indicated poor posture.^[43]^ High Intraclass Correlation Coefficient (ICC) values (0.75 and above) indicate the reliability of the NYPR. Studies have generally reported high ICC values. Additionally, the internal consistency value measured using statistical tools such as Cronbach alpha is typically 0.70 or higher, demonstrating that the tool consistently measures the same postural deviations.^[44]^ All postural assessments were performed by the same experimenter. These assessments were conducted at the beginning of each testing day, before any interventions were made, at the 4-week mark, and at the end of the 6-week follow-up period.

### 2.7. Statistical analysis

The data were analyzed using SPSS 26. Statistical significance was determined as a “*P*” value < .05. Means and ±standard deviations were calculated for continuous variables, while frequency and percentage were calculated for categorical variables. Changes in anthropometric measurements according to groups were evaluated using the independent sample *t* test for continuous variables and the chi-square test for categorical variables (Chi-square with Yates correction for 2 × 2 tables, Fisher–Freeman–Halton test for non-2 × 2 tables).

While the relevant variables showed a normal distribution in the pre-post period, both repeated measures ANOVA and generalized estimating equation (GEE) results were reported due to a high number of missing data in the follow-up phase. Repeated measures analysis of variance and the GEE method were used to determine the change by group and time. Since the number of participants decreased to 25 in the follow-up period after 6 weeks, missing observations occurred, and in this case, the GEE method was preferred over repeated measures analysis.^[45]^ An interaction test was performed during the repeated measures analysis of variance, and if it was not significant, it was reported that it was excluded from the model. When analyzed using the GEE method, it was assumed that the correlation between time points would vary; therefore, an unstructured working correlation matrix was preferred. The linear link function was used when the dependent variable was continuous, and the logit link function was used when the dependent variable was categorical.^[46]^ Clinical significance was assessed using effect size. For the classification of effect size, small (.20), medium (.50), large (.80), and very large (≥1) were used for the *d* value; small (.01), medium (.06), large (.14), and very large (.20) were used for Eta-square.^[47]^

## 3. Results

A total of 52 patients (muscle energy technique group [METG]: 26, control group [CG]: 26) completed the study after treatment. After 6 weeks of follow-up, a total of 25 patients (METG: 15, CG: 10) completed the study, while 27 patients dropped out. Reasons for withdrawal included health issues (n: 10), personal reasons (psychological and family problems) (n: 6), relocation to another city (n: 1), transportation difficulties (n: 3), and COVID-19 (n: 7). No significant harm or undesirable effects were observed in any of the groups.

Table 1 presents the socio-demographic characteristics of the patients. The demographic and clinical characteristics of the patients were not different between the 2 groups (*P* > .05).

**Table 1:** Comparison of socio-demographic characteristics (n = 52) (95% CI).

		Groups		*t*	*P*	95% CI of the difference
		METG	CG			
Age, year (mean ± SD)		53.88 ± 9.39	53.84 ± 8.24	.016*	.98	.004 [‐.539;.548]
Height, cm (mean ± SD)		163.73 ± 9.42	163.73 ± 7.70	.000*	1.0	.000 [‐.544;.544]
Weight, kg (mean ± SD)		79.42 ± 17.13	77.26 ± 14.53	.489*	.62	.136 [‐.409;.679]
BMI, kg/cm^2^ (mean ± SD)		29.65 ± 6.14	28.93 ± 5.78	.434*	.66	.120 [‐.424;.664]
Cigarette consumption, pack/year (mean ± SD)		41.70 ± 33.74	39.25 ± 31.22	.176*	.86	.075 [‐.765;.914]
Duration of pain, months (mean ± SD)		42.84 ± 32.13	33.38 ± 30.68	1.086*	.28	.301 [‐.247;.846]
				χ^2^	*P*	
Gender, n (%)	Male	11 (50)	11 (50)	.000†	1.0	
	Female	15 (50)	15 (50)			
Education level, n (%)	Primary school	13 (46.4)	15 (53.6)	1.945‡	.61	
	Secondary school	1 (25.0)	3 (75.0)			
	High school	9 (60.0)	6 (40.0)			
	University	3 (60.0)	2 (40.0)			
Smoking, n (%)	Yes	12 (57.1)	9 (42.9)	.204†	.65	
	No	14 (46.7)	16 (53.3)			
Do you exercise regularly, n (%)	Yes	1 (25.0)	3 (75.0)	.271†	.61	
	No	25 (52.1)	23 (47.9)			
FVC (% predicted) (mean ± SD)		80.92 ± 11.41	83.38 ± 11.41	.445*	‐.216	‐2 [‐8;3]
FET (s) (mean ± SD)		6.46 ± 1.45	6.42 ± 1.67	.985*	.028	0 [‐.83;.91]
FEV_1_ (% predicted) (mean ± SD)		63.53 ± 9.33	64.06 ± 8.53	.875*	‐.059	0 [‐4.40;3.30]
FEV_1_/FVC (%) (mean ± SD)		61.66 ± 5.24	61.55 ± 5.58	.949*	.021	0 [‐3.3;3.3]

BMI = body mass index, CG = control group, CI = confidence interval, FET = forced expiratory second, FEV_1_ = forced expiratory volume 1 second, FVC = forced vital capacity percent, METG = muscle energy technique group, SD = standard deviation.

* *t* test.

† Chi-square with Yates correction.

‡ Fisher–Freeman–Halton test.

### 3.1. Pain

Results showed that both treatments led to significant reductions in time-dependent pain intensity changes (at rest, during activity, and at night). Significant reductions in pain intensity at night were observed when comparing both groups after treatment and at the 6-week follow-up (*P* < .034, effect size (η2):.137) (Table 2). When the GEE results were evaluated, the pain intensity at rest after treatment was significant compared to that in the control group (*P* < .05). The decrease in pain intensity at rest after treatment in the MET group was 3.545 times higher than that in the control group. Pain intensity during activity and at night was not different in the MET group compared to that in the control group (*P* > .05) (Table 2).

**Table 2:** Changes in pain severity scores depending on group and time (*x* ± SD).

VAS (10 cm)		Group	Pretest (n(%), n = 52)	Posttest (n(%), n = 52)	Follow-up (n(%), n = 25)	Repeated measures analysis of variance for the full model (n = 25)		CG = Posttest*METG (mean ± SD, n = 52)		CG = Follow up*METG (mean ± SD, n = 52)	
						*P* ‡	η^2^	B†	*P* †	B†	*P* †
During rest	<3	METG	0 (0)	25 (96.2a)	5 (33.3)	.318	.049	3.545 (1.109)	**.001**	1.027	.243
	3–6		9 (34.6)	1 (3.8a)	10 (66.7)						
	>6		17 (65.4)	0 (0)	0						
	<3	CG	0 (0)	13 (50b)	2 (20)						
	3–6		11 (42.3)	13 (50b)	8 (80)						
	>6		15 (57.7)	0 (0)	0 (0)						
During activity	<3	METG	0 (0.0)	26 (61.9a)	6 (75.0)	.218	.064	28.303 (NC)	NC	1.319 (1.002)	.188
	3–6		12 (46.2)	0 (0.0a)	9 (52.9)						
	>6		14 (53.8)	0 (0.0)	0 (0.0)						
	<3	CG	0 (0.0)	16 (38.1b)	2 (25.0)						
	3–6		14 (53.8)	10 (100.0b)	8 (47.1)						
	>6		12 (46.2)	0 (0.0)	0 (0.0)						
During at night	<3	METG	1 (3.8)	26 (100.0a)	9 (60.0a)	**0.034**	.137	453.457 (NC)	NC	1.413 (1.324)	.286
	3–6		7 (26.9a)	0 (0.0a)	6 (40.0a)						
	>6		18 (69.2)	0 (0.0)	0 (0.0)						
	<3	CG	0 (0.0)	16 (61.5b)	2 (20.0b)						
	3–6		14 (53.8b)	10 (38.5b)	8 (80.0b)						
	>6		12 (46.2)	0 (0.0)	0 (0.0)						

CG = control group, ES = effect size based on the partial η^2^ (small, η^2^ ≥ 0.01; medium, η^2^ ≥ 0.06; large, η^2^ ≥ 0.14), follow up = end of follow-up, METG = muscle energy technique group, posttest = end of treatment, SD = standard deviation.

a,b There is a significant difference between groups with different letters, NC: not computed (could not be calculated due to convergence problem).

* Statistical significance at <.05 and are indicated in bold.

† Generalized estimation equation.

‡ Data were analyzed using repeated measure ANOVA.

### 3.2. Importance of PA

Results showed a group × time interaction for the perception of the importance of exercise, with both groups exhibiting a significantly greater increase after treatment and at the 6-week follow-up (*P* < .002, η2: .238). After treatment and at the end of the 6-week follow-up, the perception of considering PA as very important increased in the MET group compared to the control group. When the GEE results were evaluated, similar to the results of the repeated measures analysis of variance, the perception of the importance of PA in the MET group increased significantly compared to the control group after treatment and at the end of the 6-week follow-up period (*P* < .001) (Table 3).

**Table 3:** Changes in importance of physical activity, self-confidence, and posture scores depending on group and time (*x* ± SD).

		Group	Pretest (n(%), n = 52)	Posttest (n(%), n = 52)	Follow-up (n(%), n = 25)	Repeated measures analysis of variance for the full model (n = 25)		CG = posttest*METG (mean ± SD, n = 52)		CG = follow up*METG (mean ± SD, n = 52)	
						*P* ‡	η^2^	B†	*P* †	B†	*P* †
Impotance scale (score: 0–10)	0–3	METG	23 (88.5)	0 (0.0)	0 (0.0)	**.002**	.238	‐3.982 (.938)	**<.01**	‐3.331 (.907)	**<.01**
	4–6		3 (11.5)	3 (11.5a)	6 (40.0a)						
	7–10		0 (0.0)	23 (88.5a)	9 (60.0a)						
	0–3	CG	19 (73.1)	1 (3.8)	0 (0.0)						
	4–6		7 (26.9)	18 (69.2b)	10 (100.0b)						
	7–10		0 (0.0)	7 (26.9b)	0 (0.0b)						
Confidence scale (score: 0–10)	0–3	METG	12 (46.2)	0 (0.0)	0 (0.0)	**.001**	.250	‐2.293 (.609)	**<.01**	‐3.104 (1.801)	**.045**
	4–6		13 (50.0)	7 (26.9a)	4 (26.7a)						
	7–10		1 (3.8)	19 (73.1a)	11 (73.3a)						
	0–3	CG	9 (34.6)	0 (0.0)	0 (0.0)						
	4–6		17 (65.4)	19 (73.1b)	10 (100.0b)						
	7–10		0 (0.0)	7 (26.9b)	0 (0.0b)						
New York posture scale (score: 13–65)		METG	42.267 (5.007)	54.933 (5.663)	50.667 (5.499)	**<.001**	.641	7.654 (.702)	**<.001**	5.878 (.820)	**<.001**
		CG	43.200 (4.467)	47.600 (4.526)	45.400 (4.274)						

CG = control group, ES = effect size based on the partial η^2^ (small, η^2^ ≥ 0.01; medium, η^2^ ≥ 0.06; large, η^2^ ≥ 0.14), follow up = 6 weeks after posttest, METG = muscle energy technique group, posttest = 4 weeks after treatment, SD = standard deviation.

a,b There is a significant difference between groups with different letters.

* Statistical significance at <.05 and are indicated in bold.

† Generalized estimation equation,

‡ Data were analyzed using repeated measure ANOVA.

### 3.3. Self-confidence

Results showed that both groups exhibited a significant increase in the perception of self-confidence after treatment and at the end of the 6-week follow-up period (*P* < .001, η2: .250). After treatment and at the end of the 6-week follow-up, the perception of self-confidence to engage in PA increased in the MET group compared to the control group. When the clinical significance was evaluated, the effect size value was found to be large. When the GEE results were evaluated, similar to the results of the repeated measures analysis of variance, the perception of self-confidence to engage in PA in the MET group increased significantly compared to the control group after treatment and at the end of the 6-week follow-up period (*P* < .001) (Table 3). In the MET group, the perception of self-confidence to engage in PA continued to decrease compared to posttreatment.

### 3.4. Postural assessment

Results showed that both groups exhibited significant improvements in posture scores (*P* < .01). When the results of repeated measures analysis of variance were evaluated, a significant increase in posture scores was observed when comparing the METG and CG after treatment and at the end of the 6-week follow-up period (*P* < .001, η2: .641). When clinical significance was evaluated, the effect size value was found to be very large. When the GEE results were evaluated, the posture scores of individuals in the MET group increased significantly compared to the control group after treatment and at the end of the 6-week follow-up period (*P* < .001) (Table 3). It was also observed that this effect persisted at the end of the 6-week follow-up.

## 4. Discussion

This study investigated the effect of MET intervention on pain intensity, the importance of PA, self-confidence, and posture in individuals with COPD and chronic neck pain. While pain scores decreased in both groups, there was an increase in the perception of the importance of PA, self-confidence, and posture scores. However, compared to the control group, individuals in the MET group experienced a superior reduction in pain at rest and at night, with a more substantial increase in the perception of the importance of PA, self-confidence, and posture scores. The effect of the treatment continued even after 6 weeks in the MET group.

Pain is a common extra pulmonary feature in patients with COPD and is associated with a large number of specific comorbidities.^[5,7]^ The practice of MET reduces pain and muscle tone, stretches tense muscles, strengthens weak muscles, improves local circulation and provides mobilization of the limited joint.^[48,49]^ In the MET procedure, relaxation of the antagonist muscle occurs because of the active contraction of the agonist muscle. Joint mobility is facilitated by reciprocal inhibition. It has been emphasized that MET is a safe and noninvasive technique, particularly suitable for use in the upper cervical region, where more caution is typically required due to anatomical sensitivity. This is due to the fact that a resistance of 20% of the maximal contraction of the patient is imposed.^[50,51]^

Studies on pain in individuals with COPD have mostly focused on the prevalence and assessment of pain. A systematic review conducted in 2019 emphasized that they did not identify studies reporting specific intervention strategies for pain management.^[52]^ In our study, participants initially experienced moderate to severe levels of pain, as indicated by baseline pain scores (resting, activity, and night pain scores). These data are presented in Table 2. This suggests that the observed reduction in pain following the MET intervention may be due to the high pre-study pain levels, which could have led to a more pronounced improvement. Therefore, this study is novel in the literature as an intervention study specifically for pain management in individuals with COPD and chronic neck pain.

In a systematic review conducted by Morris et al in 2023, non-pharmacological and noninvasive interventions for chronic pain in individuals with COPD were examined. This study included a wide range of interventions, such as pulmonary rehabilitation, various exercise forms (aerobic exercises, resistance training, stretching movements), education, breathing management techniques, psychotherapeutic interventions, and self-management. As a result of these interventions, a clinically meaningful reduction in pain outcomes compared to baseline was reported.^[53]^ Samiullah et al investigated the effects of MET in addition to traditional physical therapy, as opposed to traditional physical therapy alone, in reducing mechanical neck pain. In their study, both groups reported a significant reduction in pain; however, it was emphasized that the reduction in pain is superior intensity was more effective in the group receiving MET.^[54]^ Gupta et al investigated the effects of post-isometric relaxation versus isometric exercises in individuals with nonspecific neck pain and reported that the MET demonstrated a significant improvement in pain and functional status.^[38]^ In our study, similar to other studies, pain intensity decreased in both groups after treatment, and the decrease in resting pain intensity of individuals in the MET group after treatment was higher than that in the pretreatment and control groups. Although pain intensity during the night did not show significant differences between the MET and control groups, a greater reduction in pain intensity at rest was observed in the MET group. This discrepancy could be due to various factors such as the nature of nocturnal pain, which may be influenced by other variables like sleep position, muscle relaxation during sleep, or the physiological effects of nighttime rest. Additionally, MET’s effects may be more noticeable during periods of activity and movement, which could explain the greater improvement observed during rest. Future studies could explore the role of nighttime pain in more detail and assess whether MET has different effects during sleep versus activity. When we consider the effectiveness of MET treatment clinically, it has a significant effect, and the effectiveness of the treatment continues to decrease in the follow-up period after 6 weeks. When clinical significance is evaluated, the effect size (rest η2: .049, activity η2: .064, night η2: .137) is found to be large. However, while these values indicate a notable effect, it is necessary to assess whether these reductions translate into meaningful improvements in patients’ daily functioning and quality of life. The neurophysiologic mechanism in MET treatment inhibits alpha motor neurons by stimulating the Golgi tendon reflex, thereby inhibiting the suboccipital muscles, reducing muscle spasm, and thus playing an important role in the reduction of pain.^[28,55,56]^

The importance of satisfaction with physical functioning has been further emphasized in recent research. These studies indicate that satisfaction with physical functioning mediates the effect of PA interventions on subjective well-being.^[57]^ Evidence suggests that low satisfaction with physical functioning is associated with greater physical impairment, increased disability in valued activities, and depressive symptoms. Additionally, positive changes in satisfaction with physical functioning have been found to mediate the beneficial effects of PA interventions on subjective well-being and are considered a significant component of health-related quality of life.^[57–61]^ Wójcicki et al emphasize that regular participation in PA positively influences individuals’ perception of the importance of PA in older adults.^[62]^ In our study, individuals in the MET group showed significant improvements in their perception of the importance of PA after treatment. This effect persisted even after the 6-week follow-up period, indicating that the changes achieved posttreatment were sustainable. From a clinical significance perspective, the effect size was found to be large, suggesting that changes in the perceived importance of PA have a practically meaningful impact. Notably, an increased perception of the importance of PA in individuals with COPD may be a crucial factor in improving their PA levels. Furthermore, these findings could play a significant role in developing treatment strategies aimed at promoting greater engagement in PA among individuals with COPD. Future research could strengthen treatment protocols by further exploring the impact of changes in the perceived importance of PA on clinical outcomes.

Individuals with COPD state that feeling safe is related to their self-efficacy. Self-efficacy refers to confidence in one’s capacity to perform certain behaviors. Elfing study suggests that generalized self-efficacy is an independent determinant of health status. Self-efficacy predicts the social and mental dimensions of health status.^[63]^ Camp et al stated that pulmonary rehabilitation training refers to the process of changing various psychosocial factors such as self-confidence. The acquisition of specific skills and knowledge of the disease by individuals with COPD assists them in managing the disease more effectively and leads to a greater sense of confidence and control over the disease.^[41]^ In our study, the perception of self-confidence in PA was significantly improved in both groups. The reason for this may be the increase in their knowledge and skills regarding the management of the disease, as well as the improvement in disease symptoms such as pain as a result of the physiological mechanism underlying MET treatment.

Pain in individuals with COPD is believed to be caused by postural abnormalities and alterations in spinal alignment. This is due to the increased workload of the respiratory muscles and hyperinflation, as well as decreased mobility of the spinal joints and costae.^[64]^ Fathollahnejad et al investigated the effect of manual therapy and stabilization exercise on 60 women divided into 3 groups with forward head and round shoulder posture; group 1 received stabilization exercise and manual therapy, group 2 received stabilization exercise, and group 3 received a home exercise program, and significant improvement in posture and pain was observed in group 1 compared to group 2.^[65]^ Joshi et al concluded that the combined effect of MET and posture correction exercises provided superior effects compared to exercises for neck range of motion and that MET should be included in the treatment of individuals with Forward Head posture.^[66]^

In our study, when we evaluated the effects of MET treatment performed on accessory respiratory muscles on posture, we observed significant improvements in posture in the MET group and discovered that these improvements remained effective after the treatment. The mechanism behind this improvement suggests that it may have a positive effect on both static and kinetic postures because of the effects on the proprioceptive and interceptive afferent pathways.^[56]^

In studies conducted on individuals with chronic musculoskeletal pain, the superior outcomes observed in the MET group compared to the control group can be attributed to MET’s direct impact on musculoskeletal dysfunction, its ability to enhance proprioceptive feedback, and its effect on reducing muscle tension. Several studies in the literature have demonstrated the effectiveness of MET in managing chronic musculoskeletal pain. For instance, Ballestero-Pérez et al conducted a systematic review and meta-analysis, highlighting MET’s effectiveness in reducing cervical muscle spasms and pain.^[67]^ Additionally, Franklin et al showed that MET significantly improved muscle flexibility and corrected postural imbalances, making it superior to conventional manual therapy for pain management.^[68]^ Similarly, Mahmoud et al reported that MET enhances proprioceptive awareness and muscle activity regulation in individuals with chronic neck pain, leading to improved long-term postural adaptation.^[69]^ Furthermore, Barden et al explored the effects of MET on respiratory function and thoracic mobility, demonstrating that this technique enhances chest expansion, which may play a crucial role in both respiratory function and musculoskeletal pain management.^[70]^ It is important to note that these control interventions likely vary across studies, and therefore, the findings should be interpreted with this variability in mind. In patients with COPD, where postural changes and respiratory muscle dysfunction are key factors in pain management, the multimodal effects of MET become even more pronounced.

The limitations of this study include the absence of a true control group that did not receive treatment, and the lack of consideration for habitual PA and medication use, which could influence the outcomes. Additionally, as the study was conducted during the COVID-19 pandemic, respiratory functions could not be assessed according to the ERS Group 9.1 recommendations. Therefore, the effect of the muscle energy technique on pulmonary function in COPD patients with chronic neck pain could not be evaluated.

## 5. Conclusion

This study revealed that MET training provided to accessory respiratory muscles was effective in reducing pain, enhancing perceived PA, improving self-confidence towards PA, and addressing postural impairment in individuals with moderate COPD and chronic neck pain. The implementation of MET therapy in addition to a treatment program may provide better benefits for the treatment of individuals in this population. Further research is required to investigate pain management in COPD patients.

## Acknowledgments

We are grateful to all individuals with COPD who agreed to participate in this study.

## Author contributions

**Conceptualization:** Mehtap Malkoç.

**Data curation:** Serpil Mihçioğlu.

**Formal analysis:** Serpil Mihçioğlu.

**Investigation:** Serpil Mihçioğlu.

**Methodology:** Serpil Mihçioğlu, Mehtap Malkoç.

**Project administration:** Serpil Mihçioğlu, Mehtap Malkoç.

**Supervision:** Mehtap Malkoç.

**Visualization:** Serpil Mihçioğlu.

**Writing – original draft:** Serpil Mihçioğlu.

**Writing – review & editing:** Mehtap Malkoç.
